# Pamidronate is ineffective for inflammatory-like low back pain associated with Modic type 1 changes: A double-blind, randomised controlled trial

**DOI:** 10.1016/j.ocarto.2026.100851

**Published:** 2026-07-23

**Authors:** Martin Soubrier, Charlotte Lanhers, Bruno Pereira, Angélique Fan, Sandrine Malochet guinamand, Marion Couderc, Agnès Lhoste, Anne Tournadre, Emmanuel Coudeyre, Sylvain Mathieu

**Affiliations:** a Rheumatology Department, CHU Clermont-Ferrand, Université Clermont Auvergne, France; b INRAE, UNH, Clermont–Ferrand, France; c Service de Médecine Physique et de Réadaptation, CHU Clermont-Ferrand, Université Clermont Auvergne, France; d Unité Neurodol, INSERM, UNH, Clermont–Ferrand, France; e Biostatistics Unit, CHU Clermont-Ferrand, Clermont-Ferrand, France; f Radiology Department, CHU Clermont-Ferrand, Clermont-Ferrand, France

**Keywords:** Inflammatory-like low back pain, Pamidronate, MRI-confirmed Modic 1 changes

## Abstract

**Objective:**

Modic type 1 (MC-1) vertebral endplate changes are associated with chronic low back pain (LBP) and often reflect an inflammatory process. Pamidronate has demonstrated analgesic effects in conditions involving bone marrow oedema. Our study aimed to evaluate the efficacy of intravenous pamidronate in patients with inflammatory-like LBP and MRI-confirmed MC-1 changes.

**Method:**

In the PEPTIDE study, a single-centre, randomised, double blind, placebo-controlled trial, adults aged 18–60 years with chronic inflammatory-like LBP unresponsive or intolerant to NSAIDs, and MRI-confirmed MC-1 were randomised (1:1) to receive either two consecutive daily infusions of pamidronate (90 mg) or placebo. The primary endpoint was the change in LBP intensity (VAS pain) after 3 months. Secondary outcomes included functional disability, quality of life, inflammatory symptoms, and analgesic consumption.

**Results:**

Forty-four participants (mean age 49.4 ± 8.9 years; 57% women) were randomised. At 3 months, the between-group difference in VAS pain reduction was not significant (difference −6.4 mm; 95%CI: −22.0 to 9.3). At 6 weeks, there was a trend toward pain improvement with pamidronate (difference −14.15 mm; 95% CI −28.85 to 0.55). Significantly more patients in the pamidronate group achieved predefined pain improvement thresholds, although this effect was not maintained at 3 or 6 months. Adverse events occurred more frequently in the pamidronate group (54.6% vs 27.3%), primarily flu-like symptoms.

**Conclusion:**

These findings are consistent with previous studies suggesting that bisphosphonates may provide a transient analgesic effect at 6 weeks but do not confer sustained clinical benefit at 3 or 6 months in MC-1-associated low back pain.

**Key points**

• Pamidronate did not significantly improve low back pain (LBP) at 3 or 6 months.
• A transient analgesic effect was observed at 6 weeks.
• No improvements were observed in disability, quality of life, or inflammatory symptoms.

## 1 Introduction

Chronic LBP is a common and debilitating condition affecting many individuals. While there are various causes of LBP, a significant proportion of patients are classified as having non-specific LBP, meaning the exact cause remains unidentified. A key development in understanding structural correlates of LBP was the description by Modic et al. of signal intensity changes in vertebral bone marrow adjacent to vertebral endplates on MRI, termed Modic changes (MC). MC represent a spectrum of vertebral endplate alterations, which may coexist or evolve over time. These alterations include: Modic type 1 (MC-1), which is indicative of bone marrow oedema and inflammation; Modic type 2, which is reflective of fatty degeneration; and Modic type 3, which is associated with subchondral sclerosis [1].

Several studies have reported an association between Modic type 1 (MC-1) changes and the presence of LBP [[2], [3], [4], [5]]. Brinjikji et al. concluded that MC-1 changes on MRI scans were more prevalent in adults with back pain (2). However, other studies found no association between the intensity of LBP and the presence of MC, suggesting that patients with MC experience no greater or lesser pain intensity than those without MC [4].

Patients with MC-1 may exhibit an inflammatory pain pattern. A cross-sectional study demonstrated that patients with MC-1 experienced longer morning pain, with the most intense pain occurring late at night and in the morning [6]. The underlying cause of MC-1 remains incompletely understood; various mechanisms have been proposed, including degenerative disc disease, trauma, low-virulence infectious discitis, and autoimmune-related inflammation [7].

Several treatments have been explored for MC-1, including steroid injections, antibiotics, physiotherapy, and biologic therapies such as infliximab, although results have been inconsistent [8,9]. While these treatments aim to address the underlying mechanisms and alleviate associated LBP, their effectiveness varies among individuals, necessitating further research [8]. MC-1 changes are characterised by increased bone turnover, inflammatory cell infiltration, and bone marrow oedema. Bisphosphonates such as pamidronate inhibit osteoclast activity and have been shown to reduce bone marrow lesions. Pamidronate has demonstrated analgesic effects in conditions involving bone oedema, including gonarthrosis, SAPHO (Synovitis, Acne, Pustulosis, Hyperostosis, and Osteitis) syndrome, and spondyloarthritis [[10], [11], [12]]. This provides a biological rationale for their potential use in MC-1-associated low back pain [13].

An exploratory study suggested potential benefits of pamidronate in MC-1-associated LBP; however, the small sample size and uncontrolled design limit interpretation [14]. More robust evidence from randomised trials evaluating bisphosphonates, including zoledronic acid, has shown inconsistent and generally transient effects [[15], [16], [17]]. Koivisto et al. concluded that 5 mg of intravenous zoledronic acid was effective in 20 patients with MC-1 changes compared to a placebo after one month, but not after one year [15]. Shea et al. also reported an improvement in pain intensity at one month in the oral zoledronic acid group (*n* = 13) at a dose of 50 mg once a week for six weeks, compared to placebo (*n* = 12), in patients with chronic LBP and Modic changes [16]. However, Cai et al. found no improvement in VAS pain after six months of 5 mg zoledronic acid in 35 patients with MC, regardless of type (1, 2, or mixed), compared to 37 patients receiving placebo [17].

Building on these preliminary insights, a single-centre randomised controlled trial was initiated to compare the analgesic efficacy of pamidronate against placebo in subjects with MC-1-associated inflammatory-like LBP.

## 2 Methods

### 2.1 Study design

The PEPTIDE study was a single-centre, double-blind, randomized, placebo-controlled trial with a parallel group design and 1:1 allocation ratio. The study protocol was registered on clinicaltrials.gov (NCT01799616) and published previously [18]. The trial received approval from the French institutional review board Comité de Protection des Personnes Sud Est VI (AU988) on 21 November 2012. We used the CONSORT reporting guideline [19] to draft this manuscript, and the CONSORT reporting checklist [20] when editing, included in supplementary file 1.

### 2.2 Patients

Participants were recruited from the Rheumatology Department of Clermont-Ferrand University Hospital (CHU Clermont-Ferrand, France) since February 2013. Inclusion criteria were: adults aged 18–60 years with inflammatory-like LBP, defined by at least one of the following: night-time awakening due to pain, morning stiffness lasting longer than 60 min, or maximal pain occurring in the morning. Symptoms were required to have been present for at least three months, with pain intensity exceeding 40 mm on the visual analogue scale (VAS). A VAS threshold of ≥40 mm was used to ensure inclusion of patients with clinically meaningful pain. Patients also had to demonstrate a lack of efficacy, intolerance or contraindications to non-steroidal anti-inflammatory drugs and/or the use of a semi-rigid back brace and/or standard conservative measures, including physiotherapy. They also had to show MRI-confirmed MC-1 changes. Dental clearance within the last six months and signed informed consent were mandatory.

Patients were excluded if they presented with significant spinal abnormalities, such as advanced disc degeneration (Pfirrmann grade IV–V), disc bulge or herniation, high-intensity zones, facet joint degeneration or effusion, spondylolisthesis, segmental instability, or major sagittal malalignment. All MRI scans were independently reviewed and validated by a musculoskeletal radiologist during a multidisciplinary meeting. Additional exclusion criteria were previous disc surgery, recent use of systemic corticosteroids or epidural/facet joint corticosteroid injections (within the past month), contraindications to pamidronate (including hypocalcaemia or severe renal impairment), prior bisphosphonate therapy, pregnancy, history of septic spondylodiscitis, current local or systemic infection, ankylosing spondylitis, fever (body temperature >38 °C), erythrocyte sedimentation rate >20 mm/h, age under 18 years, legal protection status, active psychiatric disorders, or inability to read or understand French.

### 2.3 Randomization and masking

Randomization was conducted using a computer-generated sequence with block sizes of four, stratified by sex and age categories (18–40, 40–50, and 50–60 years). Stratification by age was used to ensure balanced distribution of a potential prognostic factor across treatment groups. The randomization list was prepared by the study’s methodologist prior to trial initiation. Allocation concealment was ensured, with both patients and clinical staff blinded to treatment allocation, as were the assessors. The assessment of outcomes was performed by a physician specialising in Physical Medicine and Rehabilitation who was unaware of group assignments and potential side effects.

### 2.4 Procedures

Patients received two consecutive daily infusions of either 90 mg of disodium pamidronate diluted in 500 ml of 0.9% sodium chloride solution (total dose of 180 mg) or a placebo consisting of 500 ml of 0.9% sodium chloride solution.

To maintain blinding and minimise pamidronate-induced fever, all patients received prophylactic paracetamol. Treatments were labelled solely with the patients’ randomization numbers. Outcomes were assessed at baseline (D0), 6 weeks, 3 months (D90), and 6 months.

### 2.5 Outcomes

#### 2.5.1 Primary outcome measure

The primary endpoint was the between-group difference in LBP intensity change measured with the visual analogic scale (VAS) from baseline to 3 months.

#### 2.5.2 Secondary outcome measures

Secondary endpoints included the percentage of patients achieving a ‘’minimal important change’’ (15%–40% improvement) at 3 months [21]. Responder analyses were conducted using ≥15% and ≥40% pain reduction thresholds, consistent with previously published definitions, without implying clinical importance. These thresholds were used to reflect minimal and substantial improvement, respectively. The other secondary outcomes were functional disability, as evaluated by the Roland-Morris Disability Questionnaire (scores range from 0 to 24, with higher scores indicating greater disability) [22], and quality of life, as measured by the Dallas Pain Questionnaire which assesses daily life, vocational and recreational activities, psychological well-being, and social interaction [23]. Initially, other questionnaires (e.g. EIFEL, FABQ, MacTar and MCII/PASS) were planned, but they were not analysed due to incomplete data collection and concerns about the quality of the data.

Additional secondary measures included clinical indicators of spinal inflammation, such as nocturnal awakenings, and intensity of morning stiffness, and spinal mobility (finger-to-floor distance, Schober’s test). We also collected other baseline patients' characteristics: sex, age, body mass index and tobacco use.

Analgesic consumption was monitored through NSAID use, daily paracetamol dosage, and morphine-equivalent opioid dose according to McPherson M. [24]. Adverse events were recorded systematically to assess safety.

The 6-week assessment was included as a predefined intermediate time point for exploratory analyses and the 6-month time point was only for secondary outcomes.

### 2.6 Statistical analysis

The sample size calculation assumed a between-group difference of 30 mm on the VAS, corresponding to a large, clinically meaningful effect as previously reported [25]. It also assumed a standard deviation of 25 mm, a two-sided alpha of 0.05 and 90% power. This resulted in a required sample size of 22 patients per group. Continuous variables were presented as means ± standard deviations or medians with interquartile ranges. Normality was tested using the Shapiro–Wilk test.

Primary analyses were conducted at 3 months on an intention-to-treat basis using multiple imputation (predictive mean matching) for missing data, with imputation based on clinically relevant baseline variables (sex, age, BMI, tobacco use, and VAS pain) [26]. Ten complete data sets were imputed and results combined across imputations. The imputation model incorporated key baseline variables that were associated with both the primary outcome and treatment allocation. Due to the size of the sample, the number of variables was restricted in order to avoid overfitting. The primary outcome was analysed using the Mann–Whitney *U* test, with adjusted analyses performed via linear regression models controlling for sex and age. We decided to adjust for age and sex, as these are standard prognostic variables in studies of LBP and could potentially influence both pain perception and treatment response. Results were reported as between-group differences with 95% confidence intervals. Normality of residuals was assessed using the Shapiro–Wilk test.

Proportions of patients with clinically significant pain improvement (>15% and >40%) were compared between groups using chi-squared or Fisher’s exact tests, reporting absolute differences with corresponding 95% CIs. Adjusted analyses employed logistic regression, yielding adjusted odds ratios and 95% CIs. Secondary analyses were conducted at 6 weeks and 6 months. Longitudinal analyses incorporated repeated measures using mixed-effects models. Logistic regression models were used for responder analyses at each time point. Statistical comparisons were performed for safety outcomes, but not for medication use, as the latter was considered exploratory.

Other secondary outcomes were analysed using linear mixed models, with randomization group, time, and their interaction as fixed effects, and patient as a random effect. Residual normality was verified using Shapiro-Wilk. *P*-values and estimated differences with 95% CIs were derived from models assessing the interaction between time and randomization group, adjusting for sex and age.

The widths of the confidence intervals were not adjusted for multiplicity and should not be used for formal hypothesis testing, except for the primary outcome analysis.

Analyses were performed using Stata software (v15.0; StataCorp). A two-sided *p*-value <0.05 indicated statistical significance.

## 3 Results

**Fig. 1:**
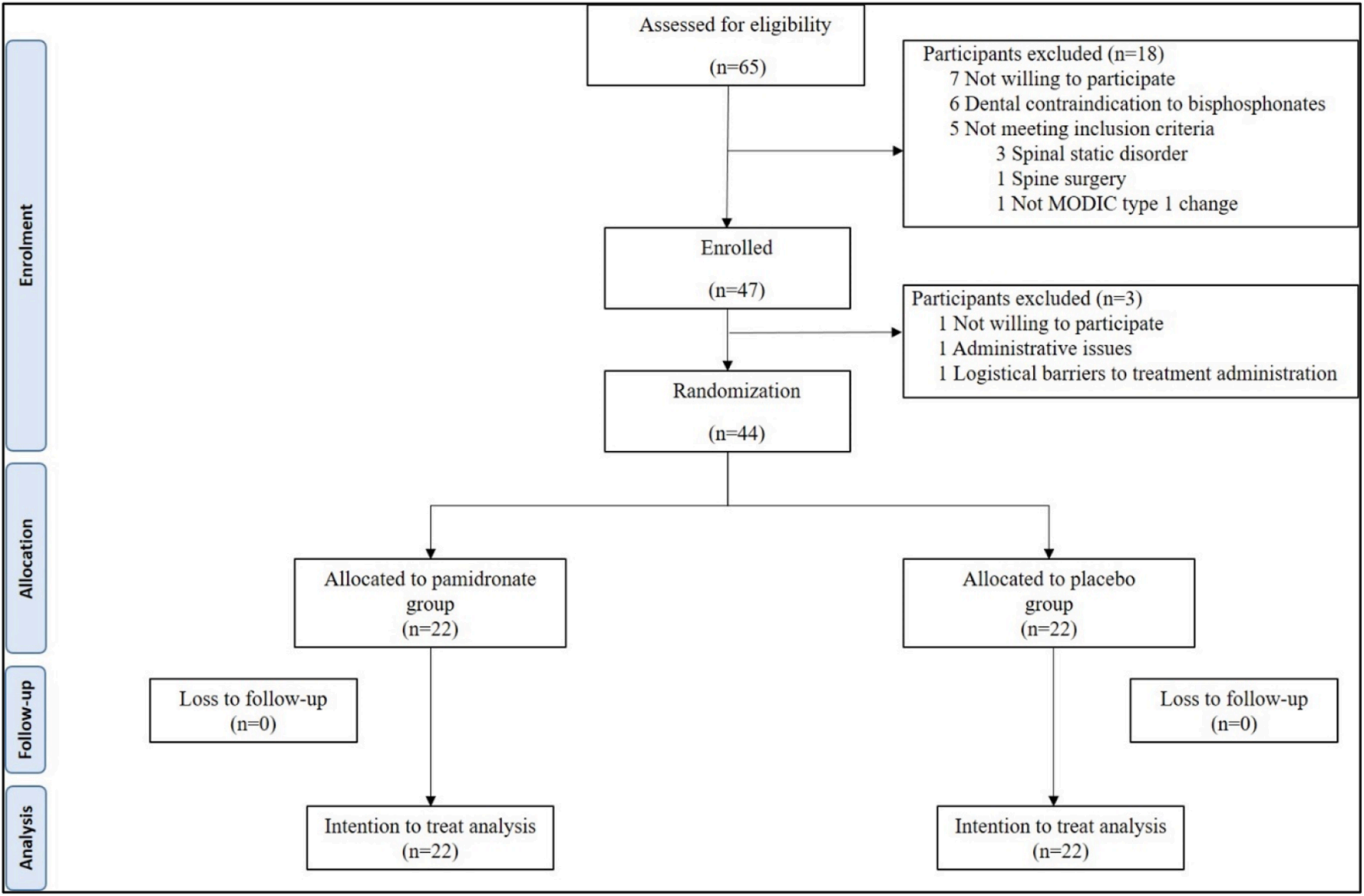
CONSORT flow diagram.

Between February 2013 and December 2019, a total of 65 patients were screened in the PEPTIDE study, of whom 47 were enrolled and 44 were randomised. Reasons for excluding three patients included withdrawal of consent, administrative issues, and logistical barriers to treatment administration (Fig. 1). Although 48 patients were initially planned, only 44 were ultimately randomised due to recruitment constraints. Patients were randomised equally into two groups: 22 in the pamidronate (PA) group and 22 in the placebo group.

The study cohort comprised 25 women and 19 men, with a mean age of 49.4 ± 8.9 years. Approximately 30% (*n* = 13) of patients were smokers. MC-1 changes were observed at the following levels: L5-S1 (*n* = 23), L4-L5 (*n* = 16), L3-L4 (*n* = 7), and L2-L3 (*n* = 2), with four patients having multi-level disc inflammation. Baseline mean LBP was 62.3 ± 15.8 mm on VAS. The mean Roland-Morris Disability Questionnaire score was 11.8 ± 15.8, and the overall Dallas Pain Questionnaire score was 28.5 ± 16.6 assessing daily activities, work and leisure, and psychological domains. Patients exhibited an inflammatory pain pattern, averaging three nocturnal awakenings and 1 h of morning stiffness. Spinal mobility was limited, with a reduced Schober index and a finger-to-floor distance of 20 cm (Table 1).

**Table 1:** Baseline participant characteristics.

	All *N* = 44	Pamidronate *n* = 22	Placebo *n* = 22
Sex Male	19 (43.2)	11 (50.0)	8 (36.4)
Age (years)	49.4 ± 8.9	50.3 ± 9.2	48.4 ± 8.7
Body mass index (kg m^−2^)	25.2 ± 3.9	25.5 ± 2.9	24.9 ± 4.7
Body mass index			
<25	19 (43.2)	8 (36.4)	11 (50.0)
25-30	19 (43.2)	11 (50.0)	8 (36.4)
≥30	6 (13.6)	3 (13.6)	3 (13.6)
Smoking	13 (30.2)	6 (28.6)	7 (31.8)
Modic at two levels	4 (9.1)	2 (9.1)	2 (9.1)
Levels of Modic change			
L2/3	2	0	2
L3/4	7	4	3
L4/5	16	7	9
L5/S1	23	13	10
Intensity of low back pain (0–100)	62.3 ± 15.8	60.1 ± 13.8	64.5 ± 17.6
Roland Morris disability questionnaire (0–24)	11.8 ± 3.5	11.4 ± 3.7	12.1 ± 3.4
Night-time awakenings	2.8 ± 2.0	3.0 ± 2.2	2.5 ± 1.7
Duration of morning stiffness (minutes)	60 [20; 90]	60 [20; 90]	45 [25; 90]
Severity of morning stiffness (0–100)	75 [60; 89]	78 [65; 90]	72 [58; 82]
Schober test (cm)	3.1 ± 1.5	3.5 ± 1.6	2.8 ± 1.3
Finger-to-floor distance (cm)	21 [4; 29]	21 [7; 35]	23 [4; 28]
Pain treatment			
Nonsteroidal anti-inflammatory drugs	16 (36.4)	8 (36.4)	8 (36.4)
Paracetamol	17 (38.6)	8 (36.4)	9 (40.9)
Posology	1000 [1000; 2000]	1900 [1000; 2913]	1000 [1000; 1000]
Opioids	17 (38.6)	9 (40.9)	8 (36.4)
Posology	10 [8; 30]	18 [5; 30]	10 [8; 30]

Data are mean ± standard deviation or median [interquartile range] or n (%).

At 3 months, both groups showed a reduction in mean LBP intensity from baseline, but the primary outcome indicated no significant difference between groups (−6.4; 95%CI −22.0 to 9.3). At 6 weeks, a non-significant trend favouring pamidronate was observed (−14.15; 95%CI -28.85 to 0.55). No differences persisted at 6 months (Table 2).

**Table 2:** Low back pain symptoms and disability at baseline, 6 weeks, 3 months, and 6 months by treatment group and as between-group comparisons (change from baseline).

	Original values		Change from baseline		Unadjusted analyses		Adjusted analyses	
	Pamidronate	Placebo	Pamidronate	Placebo	Difference (95%CI)^a^	*p*-value	Difference (95%CI)^b^	*p*-value
**Primary outcome**								
Baseline (22/22)	60.1 ± 13.8	64.5 ± 17.6	–	–	–	–	–	–
6 weeks (22/22)	46.3 ± 26.4	62.2 ± 19.8	−16 [-33; 10]	2 [-11; 13]	−14.44 (−28.58 to −0.29)	0.046	−14.15 (−28.85 to 0.55)	0.059
3 months (22/22)	45.3 ± 29.3	55.5 ± 25.4	−16 [-36; 4]	−6 [-23; 4]	−6.35 (−21.96 to 9.26)	0.416	−6.10 (−22.33 to 10.13)	0.452
6 months (22/22)	44.5 ± 26.9	44.6 ± 26.7	−16 [-37; 3]	−18 [-30; 5]	1.33 (−15.21 to 17.86)	0.872	0.69 (−16.45 to 17.84)	0.935
**Secondary outcomes**								
**Roland Morris**								
Baseline (21/22)	11.4 ± 3.7	12.1 ± 3.4	–	–	–	–		–
6 weeks (21/21)	11.1 ± 5.1	12.1 ± 3.2	0 [-2; 1]	0 [-2; 2]	0.02 (−2.36 to 2.32)	0.988	−0.02 (−2.36 to 2.32)	0.985
3 months (19/21)	9.5 ± 5.6	11.0 ± 4.1	−2 [-4; 1]	−1 [-3; 0]	−0.95 (−3.34 to 1.43)	0.434	−0.96 (−3.34 to 1.43)	0.432
6 months (17/21)	10.0 ± 5.0	10.2 ± 4.2	−2 [-4; 1]	−1 [-2; 1]	−0.40 (−2.82 to 2.02)	0.748	−0.40 (−2.82 to 2.02)	0.744
**Dallas**								
Baseline (22/22)	27.0 ± 16.4	29.9 ± 17.0	–	–	–	–		–
6 weeks (22/22)	31.4 ± 18.1	28.7 ± 18.7	1 [-1; 8]	−2 [-12; 5]	5.59 (−2.43 to 13.61)	0.172	5.59 (−2.43 to 13.61)	0.172
3 months (20/22)	25.8 ± 18.3	28.3 ± 16.3	2 [-5; 6]	−2 [-9; 6]	0.89 (−7.27 to 9.06)	0.830	0.91 (−7.25 to 9.07)	0.826
6 months (19/21)	23.2 ± 20.3	24.4 ± 15.2	−4 [-7; 4]	−5 [-20: 7]	3.16 (−5.14 to 11.45)	0.456	3.23 (−5.06 to 11.52)	0.445

	**Pamidronate**	**Placebo**	**Pamidronate**	**Placebo**	**Unadjusted** **AD (95%CI)** ***OR (95%CI)***	***p*-value**	**Adjusted** **AD (95%CI)** ***OR (95%CI)***	***p*-value**

**Pain improvement > 15%**^c^								
6 weeks (22/22)	14 (63.6)	6 (27.3)	–	–	36.4 (9.0–63.8) *4.67 (1.30 to 16.76)*	0.015	38.6 (11.5–65.7) *5.69 (1.41 to 22.9)*	0.014
3 months (22/22)	14 (63.6)	11 (50.0)	–	–	13.6 (−15.4 to 42.7) *1.75 (0.52 to 5.84)*	0.361	15.1 (−13.8 to 44.0) *1.90 (0.54 to 6.6)*	0.316
6 months (22/22)	12 (54.6)	13 (59.1)	–	–	−4.5 (−33.8 to 24.7) *0.83 (0.25 to 2.74)*	0.761	2.6 (−26.7 to 31.9) *1.13 (0.31 to 4.1)*	0.858
**Pain improvement > 40%**^c^								
6 weeks (22/22)	10 (45.5)	4 (18.2)	–	–	27.3 (0.0–53.6) *3.75 (0.95 to 14.76)*	0.052	31.2 (5.2–57.2) *5.01 (1.11 to 22.6)*	0.036
3 months (22/22)	10 (45.5)	5 (22.7)	–	–	22.7 (−4.5 to 49.9) *2.83 (0.77 to 10.43)*	0.112	20.4 (−6.9 to 47.7) *2.67 (0.69 to 10.4)*	0.155
6 months (22/22)	9 (40.9)	8 (36.4)	–	–	4.5 (−24.2 to 33.3) *1.21 (0.36 to 4.08)*	0.757	5.9 (−22.8 to 34.6) *1.29 (0.37 to 4.49)*	0.693

Data are mean ± standard deviation or median [interquartile range]. (./.) indicates number of patients for Pamidronate group/Placebo group. Original values were presented using conventional descriptive statistics (mean ± standard deviation). Change-from-baseline variables were not normally distributed and were therefore summarised using medians and interquartile ranges.

a *P*-values and estimated differences (95%CI) were obtained from a mixed model assessing the interaction between time and randomization group.

b *P*-values and estimated differences (95%CI) were obtained from a mixed model assessing the interaction between time and randomization group, adjusting for age and gender.

c For pain improvement (>15% and >40%), the results are expressed as sample size (%), absolute differences (AD) (%) (95%CI) and odds-ratio (OR) (95%CI), adjusted for age and sex in the multivariate analysis. Analyses were performed for each time endpoint (6 weeks, 3 months, and 6 months).

At six weeks, a significantly higher proportion of patients in the pamidronate group had achieved the predefined pain improvement thresholds. However, this effect was not maintained at three or six months (Table 2).

Treatment with pamidronate did not result in any significant improvements in disability as assessed using the Roland-Morris Disability Questionnaire (Table 2), quality of life as assessed using the Dallas Pain Questionnaire (Table 2), or inflammatory symptoms such as nocturnal awakenings and morning stiffness intensity and duration (Table 3). There were also no improvements in spinal mobility as measured using the Schober test or the finger-to-floor distance test (Table 3).

**Table 3:** Inflammatory characteristics and lumbar flexibility at baseline, six weeks, 3 and 6 months by treatment group and between-group comparisons of difference from baseline to 6 weeks, 3 months and 6 months.

	Original values		Change from baseline		Unadjusted analyses		Adjusted analyses	
	Pamidronate	Placebo	Pamidronate	Placebo	Difference (95%CI)^a^	*p*-value	Difference (95%CI)^b^	*p*-value
**Night waking**								
Baseline (22/22)	3.0 ± 2.2	2.5 ± 1.7	–	–	–	–	–	–
6 weeks (22/22)	2.8 ± 2.6	2.0 ± 1.6	0 [-1; 1]	0 [-2; 0]	0.14 (−1.04 to 1.31)	0.820	0.14 (−1.04 to 1.31)	0.820
3 months (20/22)	1.9 ± 2.2	2.0 ± 2.2	−1 [-2; 0]	0 [-2; 0]	−0.61 (−1.81 to 0.58)	0.315	0.61 (−1.81 to 0.58)	0.315
6 months (19/20)	2.3 ± 2.5	1.5 ± 1.7	0 [-2; 1]	−0.5 [-3; 0]	0.42 (−0.81 to 1.64)	0.503	0.42 (−0.81 to 1.64)	0.506
**Duration MS (minutes)**								
Baseline (21/22)	60 [20; 90]	45 [25; 90]	–	–	–	–	–	–
6 weeks (22/22)	40 [15; 60]	30 [15; 90]	0 [-5; 0]	0 [-10; 0]	−2.63 (−33.02 to 27.76)	0.865	−2.66 (−33.05 to 27.72)	0.864
3 months (20/21)	33 [13; 60]	30 [10; 60]	0 [-40; 5]	0 [-25; 0]	6.10 (−25.08 to 37.27)	0.702	6.10 (−25.07 to 37.27)	0.701
6 months (19/19)	30 [10; 60]	20 [5; 60]	0 [-30; 15]	−15 [-40; 0]	20.05 (−11.91 to 52.01)	0.219	20.05 (−11.90 to 52.01)	0.219
**Schober test (centimeters)**								
Baseline (21/21)	3.5 ± 1.6	2.8 ± 1.3	–	–	–	–	–	–
6 weeks (22/19)	3.4 ± 1.4	3.1 ± 1.1	0 [-1; 1]	0 [-1; 2]	−0.41 (−1.54 to 0.73)	0.481	−0.38 (−1.52 to 0.75)	0.507
3 months (20/22)	3.6 ± 3.1	3.0 ± 1.0	−1 [-2; 0]	0 [-1; 1]	−0.06 (−1.19 to 1.07)	0.916	−0.04 (−1.17 to 1.09)	0.945
6 months (19/21)	3.1 ± 1.0	3.2 ± 0.8	0 [-2; 1]	0 [0; 1]	−0.77 (−1.91 to 0.38)	0.189	−0.75 (−1.89 to 0.39)	0.199
**Finger to floor (centimeters)**								
Baseline (21/22)	21 [7; 35]	23 [4; 28]	–	–	–	–	–	–
6 weeks (22/19)	22 [10; 31]	28 [10; 34]	0 [-5; 10]	4 [0; 7]	−2.47 (−8.27 to 3.32)	0.403	−2.47 (−8.27 to 3.33)	0.404
3 months (20/22)	23 [16; 29]	24 [6; 31]	0 [-5; 8]	0 [-2; 5]	−2.02 (−7.78 to 3.74)	0.491	−2.02 (−7.76 to 3.74)	0.493
6 months (19/21)	25 [10; 34]	22 [14; 32]	0 [-6; 3]	2 [-3; 5]	−1.30 (−7.16 to 4.55)	0.663	−1.29 (−7.15 to 4.56)	0.665

MS: morning stiffness.

Data are mean ± standard deviation or median [interquartile range]. (./.) indicates number of patients for Pamidronate group/Placebo group.

a *P*-values and estimated differences (95%CI) were obtained from a mixed model assessing the interaction between time and randomization group.

b *P*-values and estimated differences (95%CI) were obtained from a mixed model assessing the interaction between time and randomization group, with adjustment on age and gender.

**Fig. 2:**
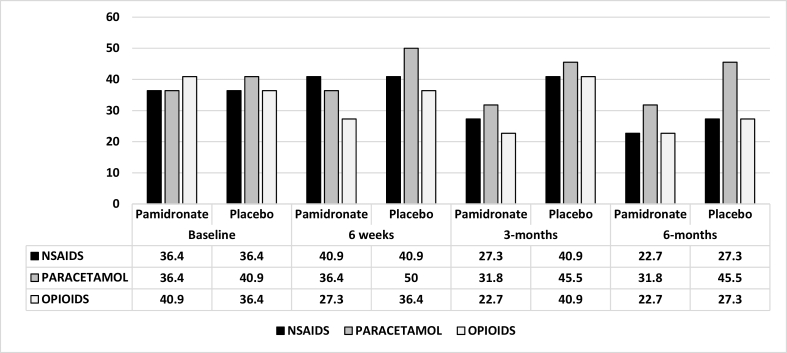
**Percentage-based evolution of NSAIDs, paracetamol and opioids consumption**. NSAIDS = nonsteroidal anti-inflammatory drugs.

At 3 months, consumption of NSAIDs, paracetamol, and opioid equivalents was substantially lower in the pamidronate group (Fig. 2).

Adverse effects, primarily flu-like syndromes, were more common in the pamidronate group (Table 4).

**Table 4:** Adverse events.

	Pamidronate *n* = 22	Placebo *n* = 22	p
Adverse events – all	12 (54.6)	6 (27.3)	0.066
Adverse events - treatment-related	11 (50.0)	3 (13.6)	0.010
Fever	3 (13.6)	0 (0.0)	0.233
Flu like symptoms	7 (31.8)	1 (4.6)	0.046
Headache	2 (9.1)	0 (0.0)	0.488
Arthralgia	1 (4.6)	2 (9.1)	1.000

Data are n(%).

## 4 Discussion

Our trial found no significant difference in pain improvement between pamidronate and placebo at the primary 3-month and secondary 6-month endpoints in patients with inflammatory-like LBP associated with MC-1 changes. A significantly higher proportion of patients in the pamidronate group achieved reductions in pain of more than 15% and 40% at six weeks. However, these differences were not sustained at later follow-up visits. No notable improvements were observed between the groups for disability (Roland-Morris Disability scores), quality of life (Dallas questionnaire), nocturnal awakenings, duration of morning stiffness, Schober test, or finger-to-floor distance. Approximately half of the pamidronate-treated patients experienced poor tolerability. We found reduced use of analgesics in the pamidronate group, which may have reduced observable differences in pain outcomes, potentially contributing to an underestimation of the effects of the treatment. However, during the trial period, three additional randomised studies investigated bisphosphonate therapy in patients with Modic discopathies. The first trial, involving 40 patients, showed a short-term reduction in LBP at 1 month with zoledronic acid (5 mg) compared to placebo, but no sustained benefit at 1 year [15]. Similarly, no improvement was noted in lower-limb pain, quality of life, work absenteeism, or lumbar flexibility, although NSAID consumption was reduced in the treatment group [15]. A second trial (*n* = 25) reported transient pain reduction at 4 weeks (5.1 ± 1.9 vs. 6.9 ± 1.8 mm, *p* = 0.038) with oral zoledronic acid (*N* = 13) (50 mg/week for 6 weeks), but no lasting benefit at 3 or 6 months, nor improvement in lower limb pain, disability, or quality of life [16]. A third trial comparing zoledronic acid (5 mg) and denosumab with placebo found no significant pain reduction at 6 months, although the Rating Scale for LBP, which separately assesses worst, average, and current pain intensity, demonstrated statistically significant improvements in both active treatment groups over 6 months compared to placebo [17]. In posthoc analyses, both medications significantly reduced VAS LBP in participants with milder disc degeneration and non-neuropathic pain, and denosumab reduced VAS LBP in those with type1 MC over 6 months, compared to placebo.

MRI-based evaluations in these trials yielded inconsistent findings. The first study, which initially had a higher prevalence of MC-1 in the zoledronic acid cohort (84.2%) than in the placebo group (50%; *p* = 0.041), reported greater conversion from MC-1 to Modic type 2 in the zoledronic acid group than in placebo (42% vs. 15%, *p* = 0.0119), with some reduction in MC-1 area [27]. In patients where MC-1 persisted throughout follow-up, the change in volume correlated positively with increased LBP intensity among placebo recipients, whereas such correlations were weaker and inverted in the zoledronic acid group [27]. The second study found no significant differences in the area of MC between treatment and control groups at baseline, 3 months, or 6 months. However, by 6 months, the zoledronic acid group exhibited a statistically significant reduction in the area of MC compared with no change in the placebo group (*p* = 0.041) [16]. The third study showed negligible overall change in MC size, with no notable differences between treatment groups, except for a subgroup receiving denosumab who had MC-1 [17]. These inconsistent findings regarding the evolution of MC on MRI scans may be due to variations in subtypes of MC and differences in follow-up intervals for imaging.

Our study has limitations. Firstly, the number of participants included may seem relatively small to draw a strong conclusion and could make the study findings less generalizable. However, with twenty-two patients per group, it was possible to detect a 30-mm pain difference on the VAS with 90% statistical power. This study was powered to detect a relatively large between-group difference in pain intensity, and therefore cannot exclude the presence of smaller treatment effects. However, the absence of consistent improvements across multiple outcomes and time points suggests that any potential benefit of pamidronate is likely to be limited and of uncertain clinical relevance. Secondly, including approximately 40 patients also corresponds with the number included in other published studies. We cannot rule out the possibility that the blind study was not perfectly adhered to, which may have impacted the results. Indeed, patients who received pamidronate experienced more flu-like symptoms, including fever and chills, despite taking paracetamol as a preventive measure. Consequently, they may have assumed that they had received pamidronate. From a methodological point of view, it would have been more rigorous to assess this potential bias by asking patients at 3 months whether they thought they had received pamidronate or the placebo. We chose to apply strict exclusion criteria to increase the homogeneity of our sample of patients with inflammatory-like LBP, but this may limit the generalisability of our findings to broader LBP populations. For instance, disc bulge or herniation, high-intensity zones, facet joint degeneration or effusion can be associated with MCs. Finally, the recruitment was slower than anticipated and the trial has been delayed approximately ten years, mainly due to the strict eligibility criteria and the requirement for both inflammatory-like LBP and MRI-confirmed MC-1 changes, which limited the pool of eligible participants.

Taken together, our study and the three additional trials suggest that bisphosphonates should not be recommended for patients with LBP associated with MC-1 changes. While some studies reported short-term pain reduction, these effects were not sustained and did not result in consistent improvements in functional disability, lower-limb pain, or quality of life. Although some studies have administered bisphosphonates orally and others intravenously, which may account for differences in pharmacokinetics, efficacy and tolerability, it nevertheless seems reasonable to conclude that bisphosphonates are not effective in treating LBP associated with MC-1 changes, regardless of their route of administration. However, given the evidence suggesting that MC-1 changes have different causes, and that there is currently no way to distinguish between these causes at an individual level, our study cannot rule out the possibility of long-term effects of pamidronate in certain subgroups of patients with these changes.

## Ethics approval and consent to participate

The trial received approval from the French institutional review board Comité de Protection des Personnes Sud Est VI (ref:AU988). All patients provided written informed consent prior to participation.

## Authors’ contributions

Pr Martin Soubrier conceptualized and designed the study, provided administrative and technical support, obtained funding, supervised data collection, interpreted data, drafted the initial manuscript, and critically reviewed and revised the manuscript.

Dr Bruno Pereira performed statistical analysis, interpreted data, drafted the initial manuscript, and critically reviewed and revised the manuscript. He performed statistical analysis, interpreted data, provided administrative support, and critically reviewed and revised the manuscript.

Ms Angelique Fan collected data and coordinated data collection, provided administrative support, and critically reviewed and revised the manuscript.

Pr Sylvain Mathieu contributed to study design, carried out data analysis and interpretation, critically reviewed and revised the manuscript.

Pr Anne Tournadre, Pr Emmanuel Coudeyre, Dr Charlotte Lanhers, Dr Sandrine Malochet-Guinamand, Dr Marion Couderc and Dr Agnès Lhoste collected data, critically revised the manuscript, and provided administrative support.

## Data availability statement

The data underlying this article may be shared on request to the corresponding author.

## Funding

This work was supported by the University Hospital of Clermont-Ferrand (ref. AOI 2012).

The study funder had no involvement in study design, data collection, analysis, interpretation, or manuscript preparation.

## Conflict of interest

All authors declare no competing interest that could influence this work.
